# Do Extracellular Vesicles Derived from Mesenchymal Stem Cells Contain Functional Mitochondria?

**DOI:** 10.3390/ijms23137408

**Published:** 2022-07-03

**Authors:** Ljubava D. Zorova, Sergei I. Kovalchuk, Vasily A. Popkov, Valery P. Chernikov, Anastasia A. Zharikova, Anastasia A. Khutornenko, Savva D. Zorov, Konstantin S. Plokhikh, Roman A. Zinovkin, Ekaterina A. Evtushenko, Valentina A. Babenko, Irina B. Pevzner, Yulia A. Shevtsova, Kirill V. Goryunov, Egor Y. Plotnikov, Denis N. Silachev, Gennady T. Sukhikh, Dmitry B. Zorov

**Affiliations:** 1 A.N. Belozersky Institute of Physico-Chemical Biology, Lomonosov Moscow State University, Moscow 119992, Russia; lju_2003@list.ru (L.D.Z.); popkov.vas@gmail.com (V.A.P.); savva@belozersky.msu.ru (S.D.Z.); roman.zinovkin@gmail.com (R.A.Z.); babenkova@belozersky.msu.ru (V.A.B.); pevzner_ib@belozersky.msu.ru (I.B.P.); plotnikov@belozersky.msu.ru (E.Y.P.); 2 V.I. Kulakov National Medical Research Center of Obstetrics, Gynecology and Perinatology, Moscow 117997, Russia; khutornenko@oparina4.ru (A.A.K.); yu_shevtsova@oparina4.ru (Y.A.S.); k_gorunov@oparina4.ru (K.V.G.); g_sukhikh@oparina4.ru (G.T.S.); 3 Shemyakin-Ovchinnikov Institute of Bioorganic Chemistry, Russian Academy of Sciences, Moscow 117997, Russia; s.kovalchuk@ibch.ru; 4 A.P. Avtsyn Research Institute of Human Morphology, Moscow 117418, Russia; 1200555@mail.ru; 5 Faculty of Bioengineering and Bioinformatics, Lomonosov Moscow State University, Moscow 119992, Russia; azharikova89@gmail.com; 6 National Research Center, “Kurchatov Institute”, Moscow 123182, Russia; konstantin.plokhikh@phystech.edu; 7 Biological Faculty, Lomonosov Moscow State University, Moscow 119992, Russia; trifonova@mail.bio.msu.ru

**Keywords:** stem cells, mesenchymal stromal cells, extracellular vesicles, exosomes, ectosomes, mitochondria

## Abstract

Extracellular vesicles (EV) derived from stem cells have become an effective complement to the use in cell therapy of stem cells themselves, which has led to an explosion of research into the mechanisms of vesicle formation and their action. There is evidence demonstrating the presence of mitochondrial components in EV, but a definitive conclusion about whether EV contains fully functional mitochondria has not yet been made. In this study, two EV fractions derived from mesenchymal stromal stem cells (MSC) and separated by their size were examined. Flow cytometry revealed the presence of mitochondrial lipid components capable of interacting with mitochondrial dyes MitoTracker Green and 10-nonylacridine orange; however, the EV response to the probe for mitochondrial membrane potential was negative. Detailed analysis revealed components from all mitochondria compartments, including house-keeping mitochondria proteins and DNA as well as energy-related proteins such as membrane-localized proteins of complexes I, IV, and V, and soluble proteins from the Krebs cycle. When assessing the functional activity of mitochondria, high variability in oxygen consumption was noted, which was only partially attributed to mitochondrial respiratory activity. Our findings demonstrate that the EV contain all parts of mitochondria; however, their independent functionality inside EV has not been confirmed, which may be due either to the absence of necessary cofactors and/or the EV formation process and, probably the methodology of obtaining EV.

## 1. Introduction

Cell therapy has become one of the most powerful strategies for the treatment of a large number of pathologies. Particular attention was paid to the introduction of mesenchymal stromal stem cells (MSC) into an organism experiencing problems caused by organ dysfunction as a result of physiological, (e.g., ischemia of different origin), chemical (drugs, poisons), biological (bacterial or viral invasion) or physical (hyperthermia, trauma) challenge. Admittedly, fetal MSC have the greatest therapeutic activity with an obvious loss of therapeutic properties as the host organism from which the MSC were isolated ages [1,2]. However, in any case, a serious limitation is the source of MSC and what is also important, obtaining it in an amount sufficient to afford the therapeutic effect, and this problem has not yet found a proper solution. So far, this problem has been mainly boiled down to the development of approaches to gross dedifferentiation of postmitotic cells, but this approach today has many limitations, primarily caused by an incomplete understanding of the mechanism of the therapeutic effect of MSC. There is an increasing number of arguments that these positive effects are not related to the differentiation of MSC introduced in the affected tissue, given the very small proportion of foreign cells compared to host cells, with obvious evidence of a positive effect [3]. This leads to the conclusion of a paracrine activity of MSC, i.e., the implanted cells introduce some chemical signals into the biological system that contribute to the survival of the affected system. Numerous speculations on the nature of these signaling substances have not yet led to some conclusions, leading to the definition of these substances as cytokines, which are provided by the secretome of stem cells. The great variety of molecules secreted by MSC makes it difficult to make a final conclusion on the universal mechanism of therapeutic action of MSC, which requires an approach to minimize the secretome system with activity equal to the activity of intact cells. One of such approaches is the therapeutic use of extracellular MSC-derived vesicles (EV) instead of intact MSC and this has been confirmed in a great number of cases, (e.g., see [4,5,6,7]). The fundamental and practical aspects of vesicle biology and medicine have become very popular topics, confirming intercellular, and maybe even inter-organ communication [8]. Much attention is paid to this communication, and in particular, the discovery of the transfer of mitochondria between cells aroused the particular interest of researchers [9,10,11,12,13,14,15] resulting in multidirectional responses of the recipient cells [16].

Recently, there have been suggestions that MSC can secrete into the external environment not only EV but also mitochondria, enabling their directional transport to cells from the microenvironment [15,17,18,19]. However, it is not entirely clear what contribution mitochondria/mitochondrial proteins make to the effects of EV derived from MSC. At the moment, there is no evidence that mitochondria, if they are entrapped in EV, can be fully functionally active, but the available data indicates the presence of mitochondrial fragments, and individual mitochondrial proteins in vesicles, although there is no data on whole organelles yet. To what extent the observed cytoprotective activity of EV preparations is due to functional mitochondria or mitochondrial proteins is unknown, since most studies are aimed at analyzing the proteome of vesicles with an emphasis on signaling or trophic factors. Thus, there are presumably two possibilities—the excretion of fully functional mitochondria separately or inside vesicles and of mitochondrial components that can activate the system through the mechanism of an innate immune response described as a response to DAMPs [20,21]. In principle, both mechanisms can provide some mobilization of the cellular system.

In this study, two fractions of MSC-derived EV were studied, differing in size and obtained under different modes of centrifugation of the MSC cultural medium. The larger vesicles with an average size of about 250 nm were called ectosomes and the smaller vesicles with an average size of about 100 nm were called exosomes. Both fractions were investigated by a variety of methods for structural and functional characteristics.

## 2. Results and Discussion

### 2.1. Electron Microscopy of EV

To address the questions raised regarding the presence of mitochondrial components in the vesicular fraction derived from MSC, we conducted a study aimed at determining the presence of mitochondrial markers in EV, with a parallel assessment of mitochondrial functional activity.

**Figure 1:**
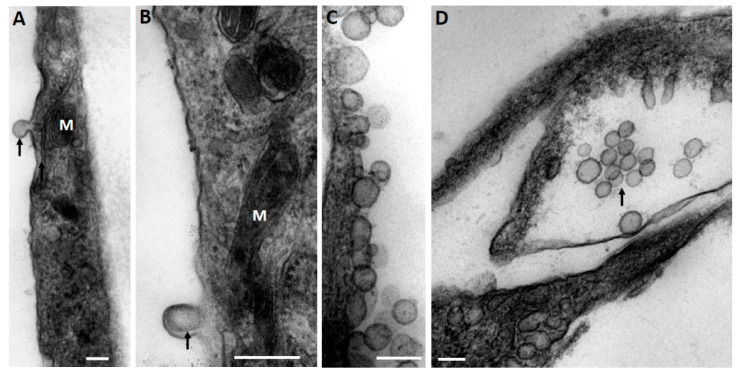
(**A**) Budding of the MSC cell membrane (shown by arrow) preferably in the future forming EV; (**B**) budding of the membrane with the presence of membranous structures inside (shown by arrow). Note the mitochondria (M) adjacent to the locus; (**C**) a group of vesicles adjacent to the cell membrane of the MSC; (**D**) a group of vesicles gathered in the intracellular space of the MSC. Electron microscopy. Scale, 250 nm.

**Figure 2:**
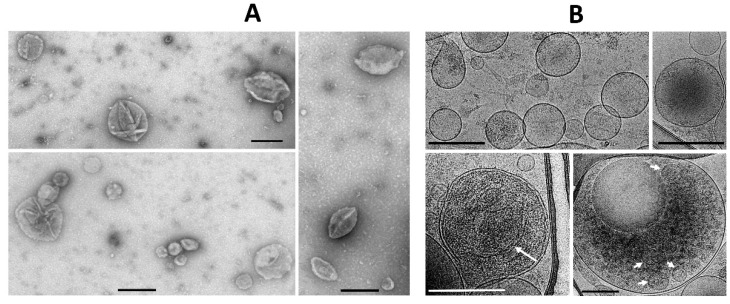
Examples of different configurations of ectosomes obtained by conventional (**A**) and cryo (**B**) electron microscopy after centrifugation of the MSC culture medium at 10,000× *g*. Note the heterogeneity of sizes with average around 250 nm, the frequent presence of shell-like profiles in (**A**), and the presence of membrane-like structures in the EV lumen in all images in A and in a simple image in (**B**) shown by a long arrow. Short arrows in (**B**) demonstrate the presence of vesicles within a multivesicular body. Bar, 250 nm in (**A**) and 300 nm in (**B**).

At the first stage, while exploring the ultrastructural characteristics of MSC, we admitted that there are grounds to assume that EV derived from MSC can be formed by two mechanisms—firstly, as a reorganization of the cell membranes through its protrusion with the capture of a part of the cytoplasm (Figure 1A), including its membrane structures (Figure 1B), not excluding capture of mitochondrial membranes. EV formation can be very powerful, as a result of which newly formed vesicles can be observed in the environment of MSC, forming clusters of EV (Figure 1C). The second rather speculative mechanism can be reduced to the intracellular formation of vesicles, as a result of which they concentrate in the form of certain depots (Figure 1D), which can subsequently be ejected from cells like the described process of mitochondria ejection from cells [13,22,23]. A key question remains: how functional are the mitochondria removed from the cell in this way? Figure 1 implies that the process of generating vesicles from MSC leads to the appearance of EV of different shapes and sizes, with a preferred size of about 250 nm, which conditionally corresponds to the size of mitochondria. Similar vesicle sizes were observed in the EV population after their isolation by centrifugation at 10,000× *g* from the medium in which the MSC were cultured with a further examination by conventional transmission electron microscopy (Figure 2A). However, extremely high heterogeneity of the vesicles was evident, both in size, shape, and ultrastructure of the vesicle lumen.

In parallel, the morphology of EV was assessed by cryo-electron microscopy, which claims to preserve membranes in a state close to the native one [24] and which has been previously used to assess the morphology of EV from cerebrospinal fluid [25].

**Figure 3:**
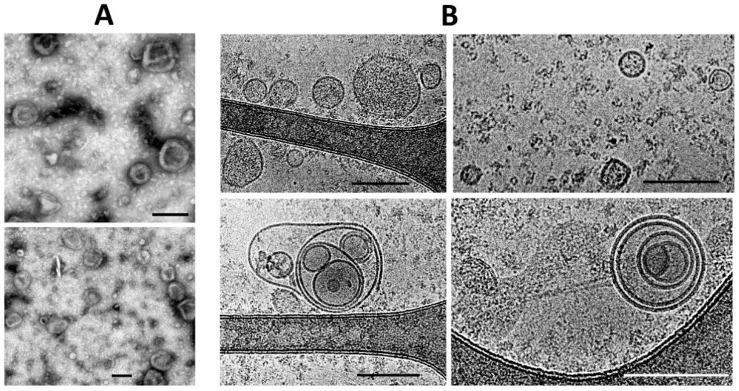
Examples of different configurations of exosomes obtained by conventional (**A**) and cryo (**B**) electron microscopy of vesicles supported on grid (grey long profiles) after centrifugation of the MSC culture medium at 108,000× *g*. Note the heterogeneity of sizes with average around 100 nm. Bar, 150 nm.

Both conventional transmission electron microscopy and cryo-electron microscopy showed similar traits both for ectosomes (Figure 2A,B) and for exosomes (Figure 3A,B). These methods indicated a high heterogeneity of the EV preparations but made it possible to characterize the average values of vesicle diameter, which was around 250 nm for ectosomes, and about 100 nm for exosomes. Membrane-like structures were confirmed by both microscopy methods in the lumen of ectosomes but not exosomes. On the other hand, the exosome population contained multi-membrane vesicles which, however, could be the result of the fusion of vesicles caused by high-speed centrifugation.

### 2.2. Heterogeneity of EV

It should be noted that theoretically, the resolution limit of flow cytofluorometry does not allow the resolution of the sizes of ectosomes (sizes up to 1–2 microns) and exosomes having a diameter of about 100 nm, forcing us to use other approaches to resolving different populations of EV.

**Figure 4:**
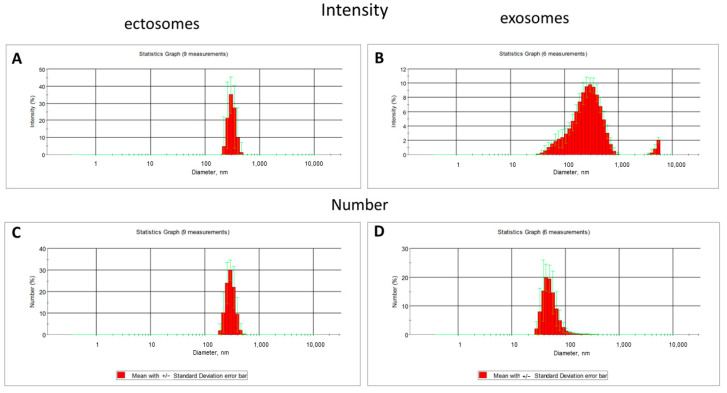
The light scattering intensity (**A**,**B**) and the numbers of particles (**C**,**D**) in two fractions of vesicles derived from MSC estimated by dynamic light scattering measurement using a Zetasizer NanoZS.

The dynamic light scattering (DLS) data indicates the relative homogeneity of the ectosome preparation (the fraction obtained by centrifugation of the culture medium at 10,000× *g*), while the fraction of exosomes was characterized by a strong heterogeneity in light scattering intensity, which may correspond to the heterogeneity of particle sizes and/or reflect some aggregation processes (Figure 4).

**Figure 5:**
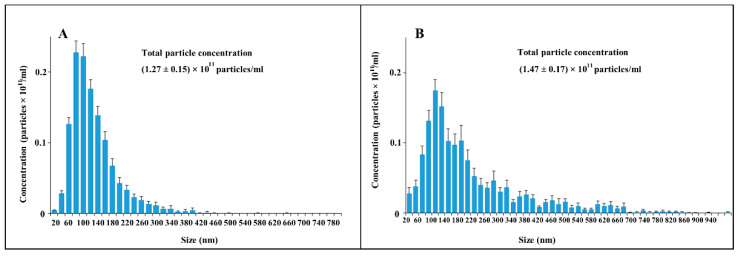
EV characterization by NTA analysis obtained in a sample either after 108,000× *g* centrifugation (exosomes, (**A**), camera gain = 500; histogram values range: 185–1885, *n* =12), or ectosomes (**B**) obtained after centrifugation at 10,000× *g* of cultivation medium (*n* =12), camera gain = 225; histogram values range: 65–1560. NTA analysis represents data of mean values with standard error of mean.

Admitting the theoretical problems of using DLS methods for EV sampling, we used nanoparticle tracking analysis (NTA), of which the results are presented in Figure 5. This approach gave similar data, but with a much wider distribution of vesicle size among ectosomes, and the mean diameters of vesicles were different (about 500 nm in the case of using the Zeta sizer and about 100 nm in the NTA analysis).

### 2.3. Interaction of EV with Mitochondrial Fluorescent Probes

**Figure 6:**
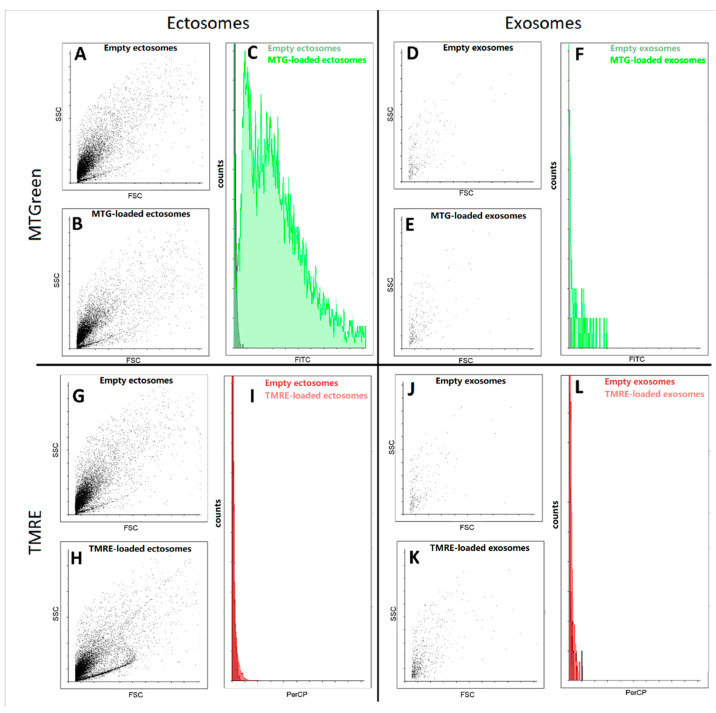
Analysis of the mitochondrial probe levels in various types of EV (ectosomes and exosomes) by flow cytometry. ((**A**–**C**,**G**–**I**), ectosomes; (**D**–**F**,J–**L**), exosomes). Dot-plots of unstained EV (empty vesicles) and EV loaded with MitoTracker Green (MTG, (**A**–**F**)) and TMRE (**G**–**L**) are presented. (**C**,**F**,**I**,**L**) Histograms of the distribution of fluorescent responses for EV loaded with MTG and TMRE on the right side of dot-plots are shown (dark green and dark red, empty EV and light green and light brown are EV loaded with MTG and TMRE correspondingly).

Further, using flow cytometry we detected fluorescence signals from samples of exosomes and ectosomes stained with mitochondrial probes MitoTracker Green and tetramethyl rhodamine ethyl ester (TMRE). We found that in ectosome samples, a subpopulation of particles stained with MitoTracker Green was observed, while exosomes isolated from the same sample were weakly stained (Figure 6). In fact, both types of particles were weakly stained, being at the error level, which does not allow us to interpret the results as evidence of the presence in the vesicles of coupled mitochondria carrying high transmembrane potential. In an additional experiment, the concentration of TMRE was increased, which made it possible to achieve pronounced staining, but this entails the danger of non-specific interactions, which also cannot unambiguously indicate the presence of maximally active mitochondria in vesicles.

In addition, the study of exosomes by this method is associated with another significant problem, since a very small number of events were detected in these samples using flow cytometry. Moreover, the detected particles did not differ in direct and side scatter of the ectosome samples. Both of these facts indicate that most likely we cannot detect the main part of exosomes due to their small size, which lies beyond the sensitivity of the cytometer detectors. Based on this, we focused our main attention on ectosomes, namely a fraction of EV obtained after centrifugation of the MSC cultural medium at 10,000× *g*.

It should be noted that mitochondria contain one unique marker which is (are) the unsaturated lipid(s) of the inner mitochondrial membranes and mitochondrial contact sites, namely cardiolipin, or rather cardiolipins, given the large set of these lipids, numbering about 100 different representatives in this group [26]. Many researchers assume that the specific staining of mitochondria with 10-nonylacridine orange (NAO) is provided precisely by the interaction of this dye with cardiolipins [27,28]; what is a simple, albeit ambiguous characteristic of the presence of cardiolipin, but what is definitely unambiguous is that NAO is a marker of mitochondria, which we used in our study.

**Figure 7:**
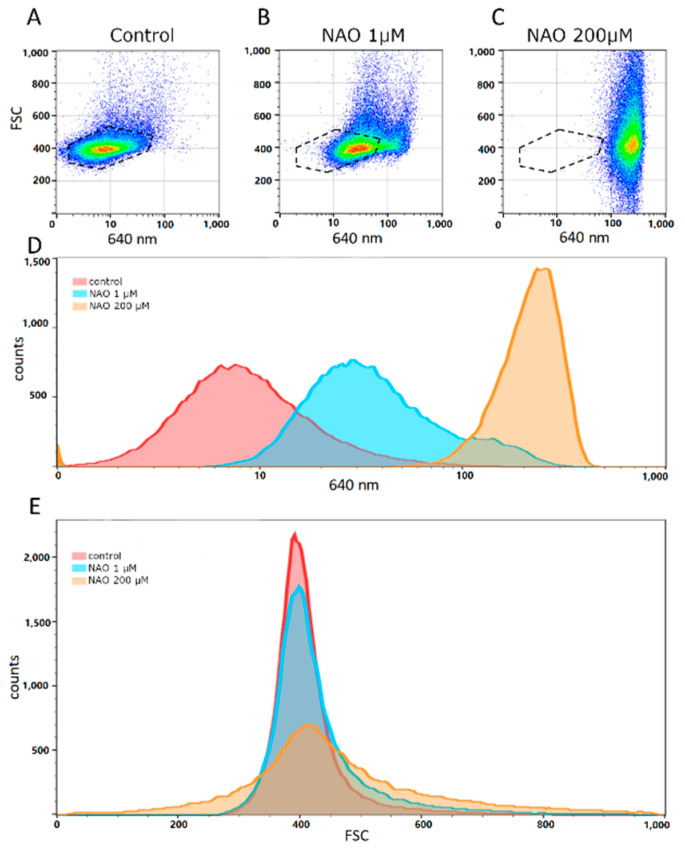
Flow cytometry analysis showing possible cardiolipin presence in ectosomes stained with 10-nonyl-acridine orange (NAO). (**A**–**C**) Size (y scale, FSC) versus fluorescent intensity (x scale, 640 nm fluorescence signal) dot-plot of control vesicles and those stained with 1 μM and 200 μM NAO. (**D**) Fluorescence (640 nm) distribution (histogram) in control vesicles and those stained with 1 and 200 μM NAO. Note the presence of a high fluorescent subpopulation in the 1 μM NAO group, which possibly indicates presence of cardiolipin. (**E**) Size (FSC) distribution in control vesicles, and those stained with 1 μM and 200 μM NAO.

Flow cytometry analysis of NAO-stained vesicles demonstrates (Figure 7A–C) that NAO fluorescence in vesicles increases in a concentration-dependent manner since the distribution shifts towards higher values with NAO concentration is clearly seen. Possibly it is due to nonspecific interactions of NAO with vesicle membrane. However, at concentration 1 μM a subpopulation of particles (seen as a shoulder on the right side of the histogram) with high fluorescence values is observed in Figure 7D, blue profile) possibly reflecting the real cardiolipin presence in vesicles. It is also worth noting that at 200 μM NAO there was a widening of forward scatter (FSC) possibly due to disturbance of lipid membranes by high concentrations of NAO (Figure 7E). Again, we do not exclude that such high concentrations of NAO may provide nonspecific binding to lipids, explaining a wider distribution of the determined vesicle sizes (Figure 7E), which indicates the appearance of very small and very large structures that were not observed in unstained vesicles. On the other hand, we also do not exclude that the negative charges of phospholipids are significantly shielded by their positive charge multiplied by mass in high concentrations, as a result of which bilayer systems are destabilized, in particular with the possibility of a fusion of vesicular structures, and maybe their fragmentation [29].

### 2.4. Mitochondrial DNA (mtDNA) Content in EV

**Figure 8:**
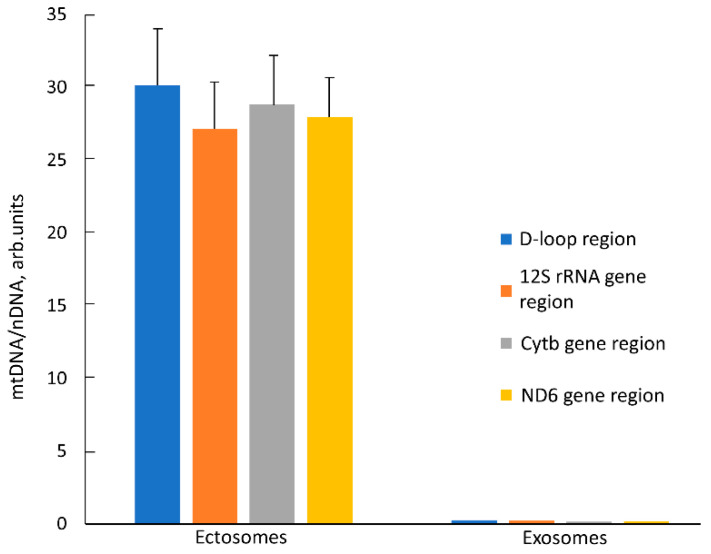
Relative mitochondrial DNA (mtDNA) content in ectosomes and exosomes. The ratio of mtDNA to nuclear DNA (nDNA) in intact MSC has been taken as 1. Four different components of mitochondrial DNA including non-coding region (D-loop) and three different gene regions were examined. Obvious enrichment of ectosomes with mitochondrial DNA (up to 30 fold) and corresponding depletion of mtDNA in exosomes (down to 0.3 fold) is shown.

Another unique marker of mitochondria is their DNA, the presence of which in itself is not proof of the presence of intact mitochondrion, but the proof of its presence in EV, together with other mitochondrial markers, is a strong argument supporting the goals of this work. We determined relative amounts of mtDNA in EV using quantitative real-time PCR (qPCR) with primers recognizing different mtDNA sites (D-loop, 12S rDNA, Cytb gene, and ND6 gene). The relative content of mtDNA vs. nuclear DNA (nDNA) in intact MSC was taken as one. In ectosomes, an ~30 times increase in the relative amount of mtDNA was observed compared to cells (Figure 8) which indicates a significant enrichment of these vesicles with mitochondria or their fragments. On the contrary, in exosomes, the relative amount of mtDNA decreased by 3–5 times relative to intact cells. We observed similar changes in the mtDNA/nDNA ratio for all pairs of primers, which indicates that these regions of the mitochondrial genome in vesicles are represented in approximately equimolar amounts. Perhaps this means that mtDNA in vesicles has not been significantly degraded, but more research is needed to verify this assumption.

### 2.5. Western Blotting of EV for Mitochondrial Proteins

In the next step, we evaluated the levels of essential mitochondrial proteins using Western blotting. We focused on proteins that participate in or regulate oxidative phosphorylation, that is, those that characterize the energy functions of mitochondria. On the other hand, we compared two populations of EV, one of which was isolated during centrifugation at 10,000× *g* (ectosomes), and for the other, the source was a supernatant obtained during centrifugation at 10,000× *g* followed by centrifugation at 108,000× *g* (exosomes) since both fractions were detected in the MSC conditioned medium possibly equally contributing to intercellular communication.

**Figure 9:**
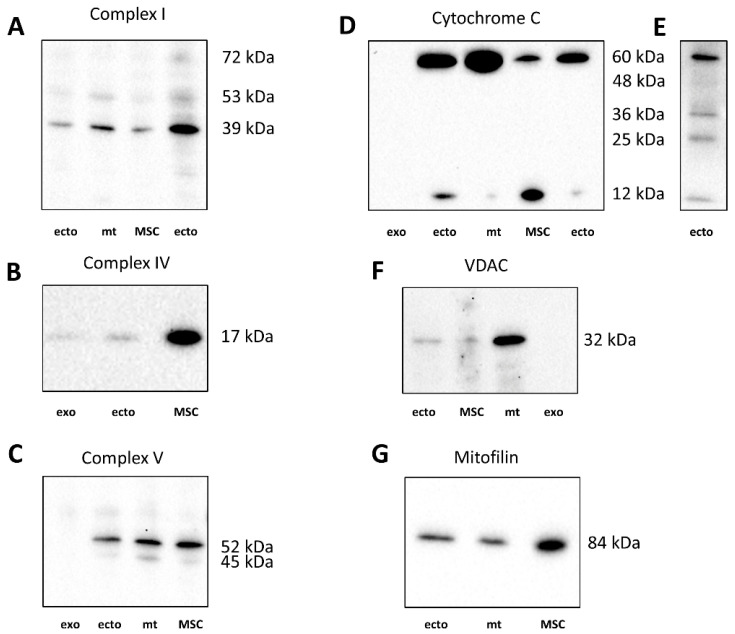
Western blots of vesicles (ectosomes, ecto and exosomes, exo), MSC, and mitochondria isolated from MSC. MWM with proper molecular masses were calculated based on molecular weight markers. For better representation of different forms of cytochrome C, the separate blot for ectosomes was made with more time exposure of the membrane. In the blot for complex I (with major 39 kDa component) in two separate lanes corresponding to ectosomes the proteins levels were different. For better demonstration of multiple forms, in the rightest lane in (**A**), the protein content of ectosomes was 4 times higher than in the very left lane (see details in Section 4 and explanations in the text). (**A**–**D**), probes for Complex I, Complex IV, Complex V and Cytochrome C, correspondingly; (**E**), example of resolution of bands for Cytochrome C in ectosomes; (**F**,**G**) probes for Complex V and Mitofilin.

A 39 kDa component of complex I was detected in ectosomes (ecto, Figure 9A), but not in exosomes (not shown). It can be noted that in addition to the target band of 39 kDa, there are additional bands with molecular masses of about 53 kDa and 72 kDa. The subunit IV of cytochrome oxidase was present in both exosomes and ectosomes preps (Figure 9B). However, the levels of this protein were significantly lower than in MSC producing these vesicles. Cytochrome C, a protein localized in the intermembrane space, on the blots, was present in five forms, which, given the molecular masses, can be attributed to mono-, di-, tri-, tetra- and pentamolecular structures (see, Figure 9E). Although it is conventional to evaluate the levels of cytochrome C by the content of its monomolecular form (about 12 kDa), we noticed that in the samples of cells, mitochondria, and vesicles, different ratios of all these forms were observed. In MSC, cytochrome C prevails in the mono-molecular form, whereas in mitochondria, the penta-molecular form prevails, and di-, tri-, and tetra were present at a very low level. In exosomes, cytochrome C was not detected in any of the forms (Figure 9D). In ectosomes, the predominance of penta- and monomeric forms with an extremely variable ratio between these two forms is mainly observed. The reason for the variability could not be identified, since this was not the purpose of this work, which was to find out whether cytochrome C is present in vesicles at all, to which a positive response was received. However, in some samples of ectosomes, the presence of bands detected at the levels probably corresponding to the di- and trimeric form was detected (Figure 9E). The presence of elements of the complex V in the heavy and light fractions of vesicles was assessed by the content of the β-subunit of ATP-synthase. In ectosome samples, this subunit (with a major band of about 52 kDa, see Figure 9C) is present, unlike exosomes. It should be noted that in the samples there was an additional band at the level of 45 kDa, which in percentage was more pronounced in the MSC mitochondria than in the MSC. Voltage-dependent anion channel (VDAC) was found in ectosomes, MSC, and mitochondria isolated from MSC but not in exosomes (Figure 9F). Mitofillin, the protein of the mitochondrial cristae, was detected in MSC and both vesicle fractions (Figure 9G).

### 2.6. Oxygen Consumption by EV Samples

Next, we evaluated the respiratory activity of vesicles using the Seahorse instrument with a proper comparison to that of mitochondria isolated from MSC.

We were unable to detect the respiratory activity of exosomes using significant variations of respiratory substrates (pyruvate + malate, succinate, TMPD + ascorbate) even with added NADH and cytochrome C to the measuring cells, believing that the availability of cofactors (in this case, NADH) or a component of the respiratory chain that can dissociate from the respiratory chain (cytochrome C) is critical. The addition of alamethicin, which permeabilizes the vesicular membrane and makes available oxidative substrates for the internal milieu of ectosomes, did not affect oxygen consumption either. Thus, we concluded that exosomes do not contain energetically competent mitochondria. 

**Figure 10:**
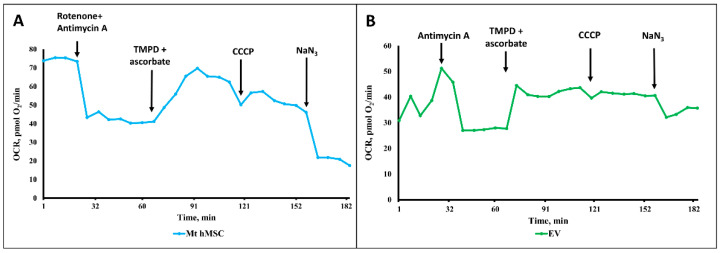
Oxygen consumption rate (OCR) of mitochondria isolated from MSC ((**A**), Mt hMSC, blue trace) and ectosomes ((**B**), EV, green trace). In (**A**), the medium contained pyruvate (10 mM), malate (2 mM), NADH (1 mM), cytochrome C (5 µM). In (**B**), the medium contained succinate (10 mM), rotenone (3 µM). Additions in ports: rotenone (3 µM), antimycin A (4 µM), TMPD (150 µM), ascorbate (10 mM), CCCP (1 µM), NaN_3_ (5 mM).

The same experiments with ectosomes gave rather contradictory results. From nine separate measurements of the respiratory activity of ectosomes, oxygen consumption was detected in five samples, while in the other four, the oxygen consumption was either very low or even negligent, or it was recorded only with the use of some oxidative substrates, but not all of those supplying electrons to all three respiratory complexes (complex I, complex II and complex IV, using pyruvate+ malate, succinate, and TMPD + ascorbate, respectively). However, careful examination of those positive cases showed that the oxygen consumption by vesicles differs in principle from oxygen consumption by mitochondria isolated from MSC (Figure 10A,B). First of all, this concerns the low sensitivity of oxygen consumption to cyanide (or azide, see Figure 10B), which in the concentrations used should completely block respiration and, accordingly, oxygen consumption. This should also apply to the action of antimycin A, which also, according to the theory of the respiratory chain hierarchy, should have completely blocked respiration on succinate. From this, it can be concluded that oxygen consumption by vesicles does not fully reflect cytochrome oxidase activity, that is, it is only partially due to the mitochondrial respiratory chain while the rest part of oxygen consumption can be referred to as the activity of other oxygen-consuming processes, i.e., oxidases other than cytochrome oxidase. Thus, the conclusion made from the measurements of oxygen consumption is that the respiratory activity of ectosomes, if any, cannot be fully attributed to the activity of mitochondria.

### 2.7. EV Proteome

To explain the low respiratory activity of ectosomes, we attempted to identify mitochondrial components using mass spectrometry.

Both exosomes and ectosomes were analyzed by bottom-up proteomics LC-MS analysis. We identified ~2000 protein groups in exosomes and ~4000 protein groups in ectosomes (the total proteome of exo- and ectosomes with proper comparison including the content of mitochondrial proteins is presented in Supplementary Table S1). For the identified protein groups, we analyzed how many of them belong to GO-CC “mitochondrion” and “extracellular exosome” terms using DAVID GO web-based tool (Table 1). The major GO CellComponents for ectosome and exosome proteomes can be found in Supplementary Table S2.

Interestingly, while both exosome and ectosome are clearly enriched in extracellular exosome proteins, being much smaller, exosomes have less available transport space and seemingly contain less protein material unrelated to exosome formation: larger ectosomes contain just 198 extra proteins related to extracellular vesicles formation in comparison to exosomes, while the number of proteins unrelated to vesicles formation increases more than three times from 895 proteins in exosomes to 2744 proteins in ectosomes. Importantly, LCMS profiles of ectosome and exosome samples have very similar intensities; thus, the difference in the number of identifications between exosomes and ectosomes is related to the protein concentration range: in exosomes, the abundance ratio between proteins related to extracellular vesicles GO CC term and other proteins are much higher than in ectosomes, so less non-exosomal proteins are identified.

The hypothesis that the ectosomes or exosomes contain intact functional mitochondria implies that all compartments of mitochondria are present in the vesicles. Mitochondria have complex structures including the outer membrane, inner membrane, matrix, and inter-membrane space. If random pieces of mitochondria find their way into the vesicles, the structure must be compromised, especially in the way of losing soluble proteins of the matrix and intermembrane space. We compared quantitatively (on the basis of intensity in LCMS data) several proteins specific to different compartments of the mitochondria between exo- and ectosomes (Table 2).

Depending on how the mitochondria get into the vesicles, various protein ratios might be expected. If intact mitochondria are supposed to enter the vesicle upon its formation, it will be impossible for too small exosomes to contain intact mitochondria, so the mitochondria structuring is lost during formation and only parts of the mitochondria can get into the vesicle, probably, mostly membrane parts. What we observed is that most of the proteins in the exosomes were ~10 times lower in abundance (normalized to the total protein composition of the sample) than in the ectosomes. On the other hand, we observed a much higher loss in matrix proteins of the Krebs cycle: while the proteins of the Krebs cycle are within an order of magnitude in concentration in ectosomes; in exosomes, some of the proteins go down just 10 times, while other proteins decrease more than 100 times and some are out of detection range. This might reflect mitochondria structure breaking so that not all the matrix space goes into the exosomes. Another interesting observation is that membrane proteins, both from the outer and inner membrane, demonstrate selectivity in their reduction in concentration in exosomes; especially the ATP synthase subunits of the inner membrane—its concentration is almost the same relative to the total protein amount in the sample for both exosome and ectosome samples. 

On the other hand, in ectosomes we observed a lot of proteins involved in normal mitochondria activity from all mitochondria compartments. We identified all proteins of the Krebs cycle, which implies that potentially it can be operational. We could see that the ectosomes contain proteins from all the original structures of the mitochondria, which can happen only if the mitochondria were transported into the ectosome intact, or the intact mitochondria were originally transported into the region of the vesicle formation with the following local breaking of the mitochondria structure with all the proteins still entering the vesicle or the mitochondria fission.

## 3. General Discussion and Conclusions

The title of this manuscript seems to require an unambiguous answer to the question: do vesicles formed by mesenchymal stromal stem cells contain functional mitochondria? This question arose from the general ideas that, firstly, the introduction of MSC into the affected biological system has a therapeutic effect [30,31,32,33,34] and, secondly, a therapeutic effect similar in direction can be obtained from the introduction of the medium in which MSC are cultivated, or its concentrate, and even better with the use of products obtained by centrifugation of the culture medium in which vesicular particles were detected [35,36]. 

It was assumed that these vesicles are a means of cellular communication [37], and the resulting reparative effect of stem cells can, at least in part, be attributed to the action of vesicles derived from them. It was logical to assume that such a method of communication (which can be directed if vehicles carry a delivery address) may be more effective than releasing signaling molecules into the environment surrounding the cell. As a result of such direct logic, the question arose: what can vesicles carry that can ensure the restoration of a remote damaged system?

On the other hand, it has been called that one of the elements of the damaged system was damage to its energetics, which are miraculously restored with the addition of stem cells or derived vesicles [38,39,40,41,42]. Again, it followed from straightforward logic that elements of the energy system, in particular mitochondria, were delivered to the affected system, which by their activity were designed to repair the damaged energy exchange, followed by the full functioning of the targeted cell and organ, as a result of which a search was undertaken for the presence of mitochondria in vesicles. Almost immediately, questions arose about the matching of the sizes of mitochondria and vesicles, the size dispersion of which in some cases did not allow the presence of mitochondria inside. Additionally, even those rare cases of demonstration mitochondrial presence inside large vesicles using electron microscopy, (e.g., [17,41,43]) did not exclude the possibility that these examples represent an early stage of lysosome formation, which added uncertainty and ambiguity to the conclusion of whether vesicles can contain functional mitochondria.

The functionality of mitochondria is uniquely determined by the presence of the transmembrane potential of hydrogen ions on their inner membrane, which is the result of the activity of proton pumps with the subsequent utilization of this potential by ATP synthase [44]. Thus, the gold standard for the functionality of mitochondria is the presence of transmembrane potential in them, which can be measured in particular using fluorescent cation probes for the membrane potential [45,46], and this was not detected in this study. Another way to detect energized mitochondria is to activate their oxygen consumption by uncouplers, protonophores that abolish the proton gradient on the inner membrane of mitochondria. In the case that at least one of these parameters could be realized, then this particular sample of mitochondria can be recognized as functionally active.

However, it must be admitted that the constant renewal of cells and mitochondria takes place in the cell, resulting in the utilization of non-functional organelles as a result of the work of the mitochondrial quality control machinery [47]. The disposal of unnecessary mitochondria may be accompanied by the release of organelles themselves from the cell or their incompletely disposed residues [48,49], as a result of which it is possible to assume the presence of mitochondrial components in the released elements. Therefore, two related scenarios can cause two different questions: (1) do vesicles contain mitochondrial markers and, if so, (2) based on data on these markers and other parameters is it possible to carry out such a reconstruction, as a result of which we can be sure of the presence of functional mitochondrial units in vesicles.

Considering extracellular vesicles as vehicles delivering signaling molecules or intact, fully functional structures, it is obvious that the minimum sizes of vesicles are sufficient to perform the first task, while in order to place whole intracellular structures in vesicles, it is necessary to ensure that the sizes of vesicles correspond to the proper structures. If we are talking about the delivery of mitochondria, then based on electron microscopic data, we must accept that the minimum size of a mitochondrion is about 100 nm. The sizes of extracellular vesicles vary very widely; therefore, in this study, two fractions were chosen to solve the goals of this work, one of which contained vesicles with an average size of 250 nm, and the other contained vesicles with an average size of 100 nm. We have examined these two fractions by a number of different methods that allowed us to evaluate the structure, content, and functionality.

Our data, based on a comparison of different sizes of vesicles by their structural and functional characteristics, did not allow us to unambiguously answer the question: do vesicles contain fully functional mitochondria? The only argument in favor of mitochondrial activity was that the oxygen consumption was partially sensitive to inhibitors of the mitochondrial respiratory chain. The rate of oxygen consumption on the substrates of the first and second complexes was low, which indicated either defects in these complexes or the absence of a number of necessary components (for example, NADH or cytochrome c). We added these components to the system, although we understood that they did not penetrate the vesicular membrane, but it could make sense if the components of the mitochondria were exposed outside the surface of the vesicles (as, for example, in the case of submitochondrial particles). However, this did not have a serious effect. Incomplete compliance with the classical views on the complete sensitivity of animal mitochondrial respiration to cyanide was the detection of cyanide-resistant vesicular oxygen consumption, which could indicate that oxygen consumption was at least partially caused by other oxidases to which TMPD donates electrons. The rate of oxygen consumption by vesicles was insensitive to the uncoupler, which indicated the absence of a proton-motive force in the system. The most significant was respiration in the presence of ascorbate and TMPD, which could indicate a deficiency of cytochrome c in the system. We must note that a similar effect of ascorbate and TMPD was found in an earlier very comprehensive study, where respiration on all substrates was extremely low with the exception of respiration on TMPD + ascorbate [42]. This demonstrates that cytochrome oxidase residing in the vesicles might be one of few functional mitochondrial factors which display its activity only when the permeable donor of electrons (TMPD) can directly interact with cytochrome oxidase bypassing cytochrome c which may be not fully functional considering its changed configuration. Western blot did detect the presence of cytochrome c in vesicles, but its configuration in vesicles was extremely variable, although it always differed from the configuration of cytochrome c in mitochondria. Extremely low levels of the 39 kDa subunit of Complex I (and in exosomes, we did not observe it at all) could explain the low respiration on the substrates of complex 1. Ectosomes contained moderate levels of the β -(catalytical) subunit of ATP synthase complex (in exosomes this subunit was not determined), mitofilin (residing mitochondrial cristae), and a voltage-dependent anion channel (residing in the outer membrane of mitochondria). With a high degree of probability, cardiolipins were present in the vesicles, which, like other components of mitochondria, can be attributed to damage-associated molecular patterns (DAMPs) [20].

This data indicates a low probability that mitochondrial components that reside in vesicles of different sizes by their presence can provide a serious contribution to changing the energetics of the recipient cell. Given the extremely low content of mitochondrial elements that are close to the levels of receptor ligands, it is preferable to assume that the mitochondrial components themselves do not carry the function to activate the energetics, and it is necessary to continue the search among other vesicle components which might be responsible for their therapeutic activity. The alternative suggestion is that mitochondrial elements entrapped in vesicles can still trigger regenerative processes through the conventional effects of DAMPs, which activate the innate immune system by interacting with toll-like receptors [20].

Based on the analysis of proteomic data, a number of important conclusions can be made. This analysis shows that, in principle, ectosomes, from the point of view of the presence of protein components, may contain components necessary for the functioning of the tricarboxylic acid cycle and membrane components involved in the energy formation process. That is, reconstruction of fully competent mitochondria seems possible. The absence of mitochondrial membrane potential, as evidenced by the low TMRE staining and insensitivity to the uncoupler of oxidative phosphorylation, is not an argument for the lack of normal mitochondrial energetics in vesicles (if any), since this is most often caused by methodological difficulties in isolating energized mitochondria, which may require the development of special additional approaches. The respiratory activity of mitochondria is not so sensitive to the methodological precautions used to preserve coupling in mitochondria, and the low oxygen consumption by vesicles, and, most importantly, the low sensitivity of oxygen consumption to cytochrome oxidase inhibitors indicates a small contribution of mitochondrial respiration to the total oxygen consumption, which could be caused by processes other than the process of electron transfer along the respiratory chain. Proteomic analysis indicates the presence in vesicles of many oxidases that may be responsible for such consumption of oxygen by vesicles; therefore, we do not exclude the possibility of participation in these processes of TMPD, which can carry out electron-donating with a final O_2_ consumption. Note that NADPH oxidase was not detected in the vesicle proteome.

We must admit that the vesicular proteome data revealed numerous proteins (such as superoxide dismutases, peroxiredoxins, thioredoxin reductases, etc.), of both mitochondrial and non-mitochondrial origin which potentially could make a significant contribution to antioxidative defense as part of the overall therapeutic efficacy of EV. 

A separate issue remains in the practical plane. Given the developing use of stem cell-derived vesicles for the treatment of a number of pathologies, (e.g., see [4,5,6,7]), the need to improve this technology becomes obvious. First, it is necessary to optimize the method of administration: intravenous, intra-arterial, intraperitoneal, or intranasal—depending on the nature and localization of the pathological challenge. Second, we need to understand the delicate mechanisms of EV interactions with cellular targets. Third, since EV carry a wide range of signaling protecting molecules having different targets, depending on the type of pathology, in the future, it is necessary to determine whether vesicles should be divided into fractions at all, and, if necessary, determine which of the fractions will be the most effective. This will allow delivering those components that will contribute to the optimal treatment of pathology without adverse and undesirable effects. Lastly, perhaps, when working in this direction, additional information will be obtained about the role of mitochondria or their components when they are enclosed in vesicles.

## 4. Materials and Methods

### 4.1. Primary Culture of MSC

Human postpartum placenta samples were obtained from healthy women 24 to 27 years old (*n* = 4) who delivered healthy full-term infants by cesarean section at the V.I. Kulakov National Medical Research Center for Obstetrics, Gynecology, and Perinatology. These women had no history of infectious diseases or pregnancy complications and were confirmed to be negative for hepatitis B virus (HBV), human immunodeficiency virus (HIV), and syphilis. The Human Research Ethics Committee of V.I. Kulakov National Medical Research Center of Obstetrics, Gynecology, and Perinatology approved the collection of human placenta samples used in the study. All protocols complied with national research guidelines. Patients provided informed written consent for the use of tissue for research purposes.

The internal part (approximately 1 cm^3^) of central placenta lobules was washed in phosphate-buffered saline (PBS) (Paneco, Moscow, Russia) several times, cut into small fragments, and enzymatically digested with 100 U/mL collagenase type I (Gibco) in serum-free Dulbecco’s Modified Eagle Medium (DMEM) (Paneco, Moscow, Russia). Cell suspensions from collagen digests were collected by centrifugation for 5 min at 300× *g* then washed in DMEM and pelleted again by 5 min centrifugation at 300× *g*. The cells were suspended in DMEM/F12 (Paneco, Moscow, Russia) (1:1) containing 7% fetal bovine serum (FBS) (Biosera, Nuaille, France) supplemented with penicillin (100 IU/mL), streptomycin (100 μg/mL) (Gibco, NY, USA), and 2 mM L-glutamine (Paneco, Moscow, Russia). Prior to use, the culture medium was centrifuged at 108,000× *g* for 1.5 h at 4 °C by an Avanti JXN-30 high-speed centrifuge (Beckman Coulter Inc., Fullerton, CA, USA) to avoid possible contamination with EV originating from FBS, then the supernatant was harvested, filtered using a bottle-top vacuum filter system with a pore size of 0.22 μm (Falcon, Corning, NY, USA), and used for further experiments as vesicle-free culture medium. Initially, cells were plated into a single 75 cm^2^ tissue culture flask (Gibco Life Technologies, Baltimore, MD, USA). Cells were incubated in a humidified atmosphere with 5% CO_2_ at 37 °C. The incubation medium was refreshed every 3–4 days to remove nonadherent cells. Cell growth and morphology were monitored daily using an inverted microscope. MSC at the third to fourth passage were used for harvesting extracellular vesicles in three-layer flasks (Nunc, Thermo Fisher Scientific, Dreieich, Germany).

### 4.2. Isolation of Extracellular Vesicles by Differential Centrifugation

To obtain EV, MSC cultures of a 3rd passage were used. EV were isolated following the guideline recommended by ISEV (International Society for Extracellular Vesicles) called MISEV2018 (Minimal Information for Studies of Extracellular Vesicles 2018) [50] using the procedure of differential centrifugation as described in [51]. Briefly, we collected two fractions of EV obtained by different regimes of centrifugation. Firstly, a conditioned medium of MSC culture at 80–90% confluence (~25 × 10^6^ cells) 24 h after medium refreshment with a total volume of 50 mL was collected and processed using serial centrifugations to remove cells and debris (400× *g* for 10 min) and followed by 10,000× *g* at 4 °C for 30 min thus giving a fraction of ectosomes. These were additionally resuspended in PBS and repeatedly centrifuged. Collected supernatants were used for exosome isolation by ultracentrifugation at 108,000× *g* for 1.5 h at 4 °C with further pellet washing with PBS followed by another spin at 108,000× *g* for 1.5 h to minimize protein contamination. The final pellets of ectosomes and exosomes were resuspended in 30 µL PBS or incubation medium (70 mM Sucrose, 220 mM Mannitol, 10 mM KH_2_PO_4_, 5 mM MgCl_2_, 2 mM HEPES, 1 mM EGTA, pH 7.2). Vesicle samples were stored at −80 °C. A resuspended pellet from nonconditioned culture medium passed through all centrifugations was used as a control sample (blank EV) to ensure that the observed effects were caused by EV from MSC and not by an unavoidable admixture of adventitious nanoparticles. The EV proteins were quantified using the Pierce BCA Protein Assay Kit (Thermo Scientific, Rockford, IL, USA) according to the manufacturer’s instructions.

### 4.3. Spatial Characteristics of Vesicles

To measure the size of vesicles obtained under various centrifugation modes, the method of dynamic light scattering was used. Using the Zetasizer NanoZS instrument (Malvern, Southborough, MA, USA) equipped with a He-Ne laser (633 nm), the effective hydrodynamic diameter of vesicles obtained at 10,000× *g* and at 108,000× *g* was evaluated. The intensity of the scattered 633 nm light was measured at 20 °C at an angle of 173°.

In addition, for quantitative analysis of EV and to avoid some artifacts of the Zetasizer Nano ZS device caused by unpredictable contributions of the presence of large vesicles to the integral scattering, we employed nanoparticle tracking analysis (NTA) of vesicles using a Nanosight LM10 HS unit (NanoSight Ltd., Amesbury, UK) as described earlier [51]. 

### 4.4. Flow Cytometry and Evaluation of Mitochondrial Functioning

EV fractions were characterized using a FACS CALIBUR flow cytometer. Staining of MSC-derived EV was performed with 1 µM MitoTracker Green (Invitrogen, Thermo Fisher Scientific, Waltham, MA, USA), 100 nM tetramethyl rhodamine ethyl ester (Molecular Probes, Eugene, OR, USA), and 1 μM or 200 μM of 10-nonylacridine orange (Molecular Probes, Eugene, OR, USA) as a mitochondria-selective fluorescent probe. Unstained EVs were used as controls. Specimens were sampled for 3 min at a flow rate of 30 µL per min and data were analyzed using Flowing Software (Turku Centre for Biotechnology, Turku, Finland).

### 4.5. EV Metabolism Analysis

Ectosomes or exosomes collected from 20 × 10^7^ MSC were added to wells of a Seahorse 8-well miniplate to 100 μL of incubation medium (70 mM Sucrose, 220 mM Mannitol, 10 mM KH_2_PO_4_, 5 mM MgCl_2_, 2 mM HEPES, 1 mM EGTA, pH 7.2) and basal oxygen consumption rate (OCR) was measured in the Seahorse XFp analyzer (Seahorse Biosciences, Billerica, MA, USA), according to manufacturer’s guidelines. Measurements were performed after injection of compounds affecting bioenergetics: carbonyl cyanide m-chlorophenyl hydrazine (CCCP) (10 μM, Sigma-Aldrich), and rotenone (2.5 μM, Sigma-Aldrich). Total measurement time was 100 min). Data analysis was performed using XFp Wave. Depending on the conditions, the measuring cell filled with ectosomes in the incubation medium contained pyruvate (10 mM), malate (2 mM), NADH (1 mM), and cytochrome c (5 µM). For mitochondria, the medium contained succinate (10 mM), and rotenone (3 µM). Additions in ports were: rotenone (3 µM), antimycin A (4 µM), TMPD (150 µM), ascorbate (10 mM), CCCP (1 µM), NaN_3_ (5 mM).

### 4.6. Transmission Electron Microscopy

MSC cultured in complete media were detached from the substrate with 0.05% Trypsin-EDTA at 37 °C and centrifuged at 300× *g*. The supernatant was removed, and the cell pellet was transferred to an Eppendorf vial. The cell pellet was resuspended and centrifuged again, supernatant was removed, and cells were fixed with 2.5% glutaraldehyde (MilliporeSigma, Burlington, MA, USA) prepared on the Sorensen phosphate buffer (pH 7.4) for 2 h at 4 °C. Then, the cell pellet was washed in 0.1 M Na phosphate buffer. Fixed samples were stained with 1% OsO_4_ in PBS, followed by dehydration in ascending acetone concentrations, stained with 1% uranyl acetate in 70% acetone during dehydration, and embedded in EPON™—Araldite mixture resin. After polymerization, 80 nm thick sections were made using the LKB Nova ultramicrotome (Stockholm, Sweden). Sections were collected on a carbon-coated Cu grid, stained with lead citrate according to Reynolds, and viewed with the electron microscope JEM-1400 (“JEOL”, Tokyo, Japan) at a 100 kV accelerating voltage.

For ectosomes analysis, a 10 μL drop of ectosomes in PBS was applied to nitrocellulose carbon-coated PELCO^®^ Cu grids (Ted Pella Inc., Redding, CA, USA) and incubated for 1 min. Liquid was removed by touching the top of the grid with filter paper. A 10 μL drop of 2% uranyl acetate was immediately applied to the grid followed by a 10 s incubation and moisture removal by touching the filter paper. Samples were examined using a transmission electron microscope, JEOL JEM-1400 TEM (JEOL, Tokyo, Japan) equipped with an Olympus Quemesa digital camera and iTEM software (Olympus Soft Imaging Solutions GmbH, Munster, Germany).

### 4.7. Cryo-Electron Microscopy

The EV samples were studied in a cryo-electron microscope Titan Krios 60–300 TEM/STEM (FEI, Hillsboro, OR, USA), equipped with a high-sensitive TEM direct electron detector (DED) Falcon II (FEI, Hillsboro, OR, USA) and Cs image corrector (CEOS, Herzogenaurach, Germany) at accelerating voltage of 300 kV. To minimize radiation damage during image acquisition low-dose mode in EPU software (FEI, Hillsboro, OR, USA) was used. Images were obtained with Falcon II DED at 18,000× and 37,000× magnification with the defocus value in the range of [−2 μm; −5 μm].

To prepare EV samples, lacey carbon grids were glow-discharged (30 s, 25 mA) in Pelco EasiGlow system. An amount of 3 μL of the aqueous solution of the sample containing exosomes and ectosomes was placed on the carbon side of grid and then blotted for 2.0 s and plunge-frozen into the precooled liquid ethane with Vitrobot Mark IV (FEI, Hillsboro, OR, USA) which resulted in embedding the samples in a thin layer of amorphous ice to preserve their native state and afford protection from radiation damage.

### 4.8. DNA Isolation and mtDNA Quantification

Total DNA from the samples was isolated using Quick-DNA Miniprep Kit (Zymo Research, Irvine, CA, USA) according to the manufacturer’s protocol. DNA was eluted by 200 µL of TE buffer. Relative mtDNA content in the samples was measured by real-time PCR analysis on the CFX96 Touch™ Real-Time PCR Detection System (BioRad, Irvine, CA, USA), using Eva Green master mix (Syntol, Moscow, Russia). PCR Cycling conditions included an initial heat-denaturing step at 95 °C for 3 min and 30 cycles at 95 °C for 15 s, 55 °C for 15 s, and 72 °C for 15 s coupled with fluorescence measurement. To confirm amplification specificity, the melting curves of PCR products were recorded from 72 to 95 °C. Each sample (5 µL) was run in triplicate, and a non-template control was added to each run. For the detection of nuclear DNA (nDNA) the primer pair was targeted to the human LINE1 retrotransposon sequence (forward, 5′-ACCTGCTCCTGAATGACTA-3′; reverse 5′-GATTCTGGTATGTGGTGTCTT-3′). For mtDNA detection, we used mitochondrial primer sets specific to: D-loop region (forward, 5′-ACCCTATGTCGCAGTATCTGTC-3′; reverse 5′-ATGATGTCTGTGTGGAAAGTGG-3′); cytochrome *b* (forward, 5′-ATGACCCCAATACGCAAAAT-3′; reverse 5′-CGAAGTTTCATCATGCGGAG-3′); 12SrRNA (forward, 5′-AGAGTAGAGTGCTTAGTTGA-3′; reverse 5′-ATGTTACGACTTGTCTCCT-3′); and ND6 (forward, 5′-CCATCGCTGTAGTATATCCA-3′; reverse 5′-TTCTTCTAAGCCTTCTCCTATT-3′). The relative amount of mtDNA for each mitochondrial primer set was calculated using the 2^−^^ΔΔCt^ method with evaluated PCR efficiencies normalized to LINE1 nuclear DNA. The mtDNA/nDNA ratio in a non-fractionated cell sample was defined as “1”. 

### 4.9. Western Blotting

Samples of vesicles and mitochondria were mixed with a 4-fold sample buffer containing 0.125 M Tris-HCl (pH 6.8), 4% sodium dodecyl sulfate, 40% glycerol, 0.05% bromophenol blue, and 10% 2-mercaptoethanol, boiled for 5 min, and then used for immunoblotting. Samples were loaded on 15% Tris-glycine polyacrylamide gels. After electrophoresis, gels were blotted onto polyvinylidene difluoride (PVDF) membranes (Amersham Pharmacia Biotech, Amersham, UK). Membranes were blocked with 5% blocking agent (Amersham Pharmacia Biotech, RPN2125V, Amersham, UK) in PBS containing 0.05% Tween-20, and subsequently incubated with primary antibodies: anti-OxPhos Complex I 39 kDa subunit 1:1000 mouse (A21344, Molecular probes, Eugene, OR, USA), anti-cytochrome oxidase subunit IV 1:1000 mouse (A21348, Invitrogen, Waltham, MA, USA), anti-ATP Synthase subunit B clone 11/27-7-A8 1:1000 mouse (MABS1304, EMD Millipore Corp., Burlington, MA, USA), anti- cytochrome C 1:1000 mouse (#556433, BD Pharmingen, Franklin Lakes, NJ, USA), anti-VDAC 1:1000 rabbit (#4866, Cell Signaling, Danvers, MA, USA), anti-mitofilin 1:1000 mouse (M04102, Boster Biological Technology, Pleasanton, CA, USA). Then, the membranes were incubated with secondary antibodies: anti-rabbit IgG or anti-mouse IgG conjugated with horseradish peroxidase 1:7500 (Jackson ImmunoResearch, Farmington, CA, USA) and detected using Advansta Western Bright ECL kit (Advansta, San Jose, CA, USA) using V3 Western Blot Imager (BioRad, Hercules, CA, USA). Protein concentration was measured using a commercial kit based on bicinchoninic acid (MilliporeSigma, Burlington, MA, USA).

### 4.10. Isolation of Mitochondria

MSC were washed with PBS, dissociated from flasks with 0.05% Trypsin-EDTA (Paneco, Russia), and precipitated by centrifugation at 400× *g* for 5 min, and the resulting pellet was resuspended in cooled medium for mitochondrial isolation (150 mM KCl, 5 mM HEPES, pH 7.4). Next, the cells were homogenized and sequentially centrifuged at 1200× and 8000× *g* (at 2 °C), respectively.

### 4.11. Proteome Analysis

EV were washed with PBS buffer and lysed by sonication in the minimum workable volume of 30 µL of the lysis buffer containing 1% SDS, 100 mM Tris, pH 8.5 with MS-SAFE protease inhibitor cocktail (MilliporeSigma, Burlington, MA, USA). TCEP and CAA were added to the final concentrations of 10 and 20 mM, respectively, and Cys reduction and alkylation was achieved by 10 min heating of the samples at 70 °C. 0.5 µg of trypsin (Promega, Madison, WI, USA) was added and the digestion was carried out at 37 °C overnight. Proteolysis was stopped by adding TFA to 1%. Precipitated SDC was removed by centrifugation. The samples were loaded to LC-MS directly without SPE.

LC-MS analysis was carried out on an Ultimate 3000 RSLCnano HPLC system connected to a QExactive Plus and QExactive HFX mass spectrometers (Thermo Fisher Scientific, Waltham, MA, USA). Samples were loaded to a home-made trap column 50×0.1 mm, packed with Inertsil ODS3 3 µm sorbent (GL Sciences, Tokyo, Japan), in the loading buffer (2% ACN, 98% H_2_O, 0.1% TFA) at 5 µL/min flow rate for 5 min and separated at RT in the fused-silica capillary column 300 × 0.1 or 500 × 0.1 mm (Molecta, Moscow, Russia) packed with Reprosil PUR C18AQ 1.9 (Dr. Maisch, Germany) into the emitter prepared with P2000 Laser Puller (Sutter, Novato, CA, USA) [52]. Samples were eluted with a linear gradient of 80% ACN, 19.9% H_2_O, 0.1% FA (buffer B) in 99.9% H_2_O, 0.1% FA (solvent A) from 4 to 36% of solvent B in 1 h at 0.44 µL/min flow at RT. 

MS data were collected in DDA mode. MS1 parameters were as follows: 70 K resolution, 350–2000 scan range, max injection time 50 ms, AGC target 3 × 10^6^. Ions were isolated with 1.4 m/z window and 0.2 m/z offset targeting 10 highest intensity peaks of +2 to +6 charge, 8 × 10^3^ minimum AGC, preferred peptide match, and isotope exclusion. Dynamic exclusion was set to 40 s. MS2 fragmentation was carried out in HCD mode at 17.5 K resolution (QExactive Plus) and 30 K resolution (QExactive HFX) with 27% NCE. Ions were accumulated for max 45 ms with target AGC 1 × 10^5^. 

Raw spectra were processed using in MaxQuant 1.6.6.0 (MQ) [53] and Perseus [54]. The data were searched against the Human Uniprot SwissProt database, containing canonical proteins, version from 2019 10 03. MaxQuant search was performed with the default parameter set, including Trypsin/p protease specificity, max 2 missed cleavages, Met oxidation, Protein N-term acetylation, and NQ deamidation as variable modifications, and Carbamidomethyl Cys as a fixed modification, max 5 modifications per peptide, 1% PSM and protein FDR. The following options were turned on: second peptide, maxLFQ, and match between runs. All runs were analyzed as independent experiments.

Identified protein groups were further analyzed by using DAVID [55,56], GO, KEGG, Reactome, and DOSE tools. The participation of mitochondrial proteins identified as part of the BB-MSC proteome in signaling pathways and biological processes identified using GO, KEGG, Reactome, and DOSE was evaluated.

## Figures and Tables

**Table 1:** Mitochondrion and extracellular exosome GO CC term enrichment in proteins identified in the exosome and ectosome samples. Full human proteome composition for the same terms is shown for comparison.

Protein List	Total Proteins	Proteins Assigned to GO CC Term (Number of Proteins/% of Total)		
		Extracellular Exosome	Other than Extracellular Exosome	Mitochondrion
UniProtKB-SwissProt (Human)	20,376 (100%)	2143 (10.5%)		1376 (6.75%)
Ectosome proteome	3840 (100%)	1096 (28.5%)	2744 (71.5%)	681 (17.7%)
Exosome proteome	1783 (100%)	888 (49.8%)	895 (50.2%)	186 (10.43%)

**Table 2:** Protein composition comparison between exo- and ectosome for proteins belonging to different mitochondria compartments.

Protein UniProt Identifier	Protein Name	Gene Name	Intensity * in Ectosomes	Intensity in Exosomes
**Outer Membrane**				
P21796	Voltage-dependent anion-selective channel protein 1	VDAC1	1.99 × 10^10^	2.90 × 10^9^
O96008	Mitochondrial import receptor subunit TOM40 homolog	TOMM40	1.27 × 10^9^	n/a
O94826	Mitochondrial import receptor subunit TOM70	TOMM70A	1.41 × 10^9^	n/a
**Intermembrane space**				
P99999	Cytochrome c	CYCS	1.82 × 10^9^	3.08 × 10^8^
P54819	Adenylate kinase 2, mitochondrial	AK2	8.51 × 10^8^	n/a
P00568	Adenylate kinase isoenzyme 1	AK1	5.27 × 10^8^	1.64 × 10^8^
Q9Y6K8	Adenylate kinase isoenzyme 5	AK5	1.98 × 10^8^	n/a
**Inner membrane**				
P06576	ATP synthase subunit beta, mitochondrial	ATP5B	3.83 × 10^10^	5.23 × 10^9^
P25705	ATP synthase subunit alpha, mitochondrial	ATP5A1	3.35 × 10^10^	6.00 × 10^10^
P13073	Cytochrome c oxidase subunit 4 isoform 1, mitochondrial	COX4I1	2.31 × 10^9^	3.42 × 10^8^
Q16795	NADH dehydrogenase [ubiquinone] 1 alpha subcomplex subunit 9, mitochondrial	NDUFA9	1.48 × 10^9^	n/a
P24539	ATP synthase F(0) complex subunit B1, mitochondrial	ATP5F1	3.79 × 10^9^	1.02 × 10^8^
**Matrix**				
P40926	Malate dehydrogenase, mitochondrial	MDH2	1.53 × 10^10^	1.05 × 10^9^
O75390	Citrate synthase, mitochondrial	CS	6.93 × 10^9^	2.84 × 10^8^
Q02218	2-oxoglutarate dehydrogenase, mitochondrial	OGDH	5.88 × 10^9^	1.17 × 10^7^
Q99798	Aconitate hydratase, mitochondrial	ACO2	5.62 × 10^9^	7.05 × 10^7^
P31040	Succinate dehydrogenase [ubiquinone] flavoprotein subunit, mitochondrial	SDHA	4.10 × 10^9^	n/a
P07954	Fumarate hydratase, mitochondrial	FH	3.39 × 10^9^	2.33 × 10^8^
O43837	Isocitrate dehydrogenase [NAD] subunit beta, mitochondrial	IDH3B	1.87 × 10^9^	n/a
P21912	Succinate dehydrogenase [ubiquinone] iron-sulfur subunit, mitochondrial	SDHB	1.30 × 10^9^	n/a
Q99643	Succinate dehydrogenase cytochrome b560 subunit, mitochondrial	SDHC	2.37 × 10^8^	n/a
O14521	Succinate dehydrogenase [ubiquinone] cytochrome b small subunit, mitochondrial	SDHD	1.73 × 10^8^	n/a
Q9NX18	Succinate dehydrogenase assembly factor 2, mitochondrial	SDHAF2	3.07 × 10^6^	n/a

* ”MaxLFQ Intensity” values are listed for ectosomes and “Intensity” values are listed for exosomes, obtained from MaxQuant quantitative LC-MS data analysis.

## Data Availability

Not applicable.

## Supplementary Materials

The following supporting information can be downloaded at: https://www.mdpi.com/article/10.3390/ijms23137408/s1.
